# Rates and determinants of neonatal mortality in two rural sub-districts of Sylhet, Bangladesh

**DOI:** 10.1371/journal.pone.0206795

**Published:** 2018-11-21

**Authors:** Gulam Muhammed Al Kibria, Rasheda Khanam, Dipak Kumar Mitra, Arif Mahmud, Nazma Begum, Syed Mamun Ibne Moin, Samir Kumar Saha, Abdullah Baqui, Projahnmo Study Group in Bangladesh

**Affiliations:** 1 Department of Epidemiology and Public Health, University of Maryland Baltimore, Baltimore, MD, United States of America; 2 Department of International Health, Johns Hopkins Bloomberg School of Public Health, Baltimore, MD, United States of America; 3 International Center for Maternal and Newborn Health, Department of International Health, Johns Hopkins Bloomberg School of Public Health, Baltimore, MD, United States of America; 4 Independent University, Dhaka, Bangladesh; 5 Department of Microbiology, Dhaka Shishu Hospital, Dhaka, Bangladesh; Medical Research Council, SOUTH AFRICA

## Abstract

**Introduction:**

Reducing neonatal mortality rate (NMR) is a challenge in many low- and middle-income countries including Bangladesh. In 2014, the estimated NMR in this country was 28 per 1,000 live births. This rate is higher in rural regions compared to the national average. Currently, Sylhet Division has the highest NMR in Bangladesh. Investigating rates and determinants of neonatal mortality in rural regions of this high-risk division is particularly important to implement evidence-based programs. This study examined rates and determinants of neonatal deaths in a large rural cohort in Sylhet Division.

**Methods:**

We analyzed data from a multi-country cohort study, Aetiology of Neonatal Infections in South Asia. From November 2011 to December 2013, this study was conducted in two rural sub-districts in Sylhet Division. Community health workers followed 28,960 pregnant women and their newborns up to two months postpartum and collected data on pregnancy outcomes and newborns’ survival status. The NMR was obtained by dividing total number of neonatal deaths with all studied newborns. Logistic regression was employed to calculate adjusted odds ratios (AORs) and 95% confidence intervals (CIs) for factors associated with neonatal mortality. Stata 14.0 was used for data analysis.

**Results:**

This study analyzed data of 21,227 newborns. The NMR was 43.4 (95% CI: 39.3–48.0) per 1,000 live births (N = 922). Multivariable analysis showed that the odds of neonatal mortality were significantly higher among male newborns (AOR: 1.5, 95% CI: 1.2–1.8), babies born before 34 weeks of gestation (AOR: 5.0, 95% CI: 4.1–6.1), those who were twins or triplets (AOR: 6.2, 95% CI: 3.6–10.9), and first-born child (AOR: 2.9, 95% CI: 1.6–5.3). Additionally, maternal age 30–35 years (AOR: 1.4, 95% CI: 1.-1.8), history of child death (AOR: 1.6, 95% CI: 1.2–2.2), and delivery complications (AOR: 2.1, 95% CI: 1.6–2.6) had positive associations with neonatal deaths.

**Conclusion:**

Public health programs in Bangladesh need to adopt a comprehensive strategy to address the individual, maternal, and intrapartum factors associated with neonatal mortality in rural regions. Interventions should aim to prioritize managing pre-term deliveries, first-born child, and delivery complications among pregnant women.

## Introduction

Globally, an estimated 5.9 million under-five children died in 2015. About 45% of these babies died during the neonatal period (i.e., within first 28 days of life) [1,2]. Though under-five mortality declined by 53% between 1990 and 2015, the neonatal mortality declined slower (47%) than the reduction of post-neonatal under-five mortality (58%) within the same period [1,2]. In 2015, about 98% of neonatal deaths occurred in developing countries, and South Asian countries experienced 40% of these deaths [2]. Most countries in this region have a high neonatal mortality rate (NMR), such as Bangladesh, whose NMR reached about 28 per 1,000 live births in 2014 [2,3].

The Millennium Development Goal 4 (MDG-4) aimed to reduce under-five mortality by two-thirds between 1990 and 2015; many countries failed to achieve this target due to the slower NMR reduction [1,2]. Recognizing the importance of stagnating NMR, the Sustainable Development Goals (SDGs) explicitly stated the need to reduce it. The SDG 3.2 aims to reduce the NMR to less than 12 per 1,000 live births in all countries by 2030 [4]. Although Bangladesh achieved the MDG-4 target by reducing the under-five mortality rate to 46 per 1,000 live births, the progress of NMR reduction has been slower. For instance, from 1990 to 2015, the post-neonatal under-five mortality rate declined by 71% while the NMR decreased by only 46% [3]. In 2014, more than 60% of under-five children deaths in Bangladesh occurred among neonates [3]. Therefore, accelerating the pace of progress for reducing neonatal deaths is vital to achieve the NMR target of SDGs.

Previous studies have concluded that multiple factors simultaneously affect NMR in developing countries including Bangladesh [5–10]. Infants’ individual characteristics such as lower gestational age and gender are associated with greater neonatal deaths [11–13]. Maternal complications during, before, or after delivery, as well as socioeconomic status of the family are also important determinants of neonatal mortality. Prevalence of these risk factors is disproportionately higher in developing countries including Bangladesh; the observed higher NMR results from this disproportionate prevalence in resource-limited settings [5–10].

Studies have also shown that an increased coverage of currently available, feasible, and cost-effective interventions like cord cleansing with chlorhexidine, kangaroo mother care (KMC) or antenatal corticosteroids could reduce about three-fourths of neonatal deaths [14,15]. Identifying risk factors and addressing them through a package of evidence-based interventions are essential to avoiding these unwanted and preventable deaths. Understanding the factors associated with neonatal mortality in rural areas is of particular importance to reduce the high NMR in Bangladesh since over 65% of the population lives in these regions [16]. Moreover, in 2014, the estimated NMR was higher in rural areas (31 per 1,000 live births) in this country compared to urban areas (21 per 1,000 live births) and to the national average (28 per 1,000 live births). Besides this rural-urban difference, a wide regional or divisional variation has also been observed in NMR estimates. For instance, in 2014, the estimated NMR in Sylhet division was 39 per 1,000 live births, while it was 21 per 1,000 live births in Barisal [3]. Although many studies have investigated the rates and risk factors for neonatal or childhood mortality in Bangladesh, there has been limited research about such determinants in rural Sylhet. This lack in epidemiologic literature limits our understanding of the factors to prioritize for an evidence-based programming in this high-risk region of neonatal survival [9,17–22].

Using data from a community-based cohort study conducted in rural Bangladesh, we examined the rates and determinants of neonatal mortality. We assessed the association of individual, maternal, intrapartum, household, and socioeconomic factors with neonatal deaths. Our study may find relevant applications to design interventions aimed at reducing neonatal mortality in low-resource settings.

## Methods

### Study design

This analysis used data from a study known as Aetiology of Neonatal Infections in South Asia (ANISA). ANISA is a multi-country cohort study conducted in Bangladesh, India, and Pakistan with a goal of providing data for designing appropriate treatment regimens and prevention strategies to reduce the burden of bacterial and viral infections in neonates and young infants. Details of this study have been described previously [23,24].

### Study settings and population

The Project for Advancing Health of Newborns and Mothers (Projahnmo) research group implemented the ANISA study in two rural sub-districts (Zakigang and Kanaighat) of Sylhet district under Sylhet division, located in the north-eastern part of Bangladesh [23,25]. The study site is about 300 km away from Dhaka (i.e., the capital city of Bangladesh) and 50 km away from Sylhet city (i.e., the divisional headquarter of Sylhet). The surveillance took place from November 2011 to December 2013. The estimated population in these two sub-districts is about 350,000. Most of the males are agricultural workers and the females are usually housewives. People in each of the sub-districts are served by one upazilla (i.e., sub-district) health complex located in the head-quarter of each sub-district [23]. Most of the women deliver at home aided by unskilled attendants [23,26]. A large proportion of sick newborns do not receive care from skilled health care providers in this area [20,21].

### Study implementation

Community health workers (CHWs) made two-monthly home visits to identify pregnancies. They identified 28,960 pregnant women. After explaining the study procedure, CHWs invited the pregnant women to participate and obtained verbal consent from the women or the household heads. For this part, each participant or the household head provided verbal consent, as this part of the study did not involve any invasive procedures. The waiver of written consent for this part had no adverse impact on the rights or welfare of participants. ANISA study components with invasive procedures such as blood collection from newborns required written informed consent from the primary caregiver [23,24].

CHWs then visited the consented mothers twice during the pregnancy period. They also provided a package of maternal and newborn health interventions that included counseling and education on preventive care during pregnancy and on maternal and neonatal danger signs requiring referral for emergency care during antepartum, intrapartum, and postpartum periods. The CHWs conducted the first visit immediately after confirming pregnancy and then attempted to make a second visit in the 29^th^ week. Household, socio-economic, demographic, and previous pregnancy data were collected on the first visit. CHWs attempted to identify newborns immediately after birth and visited them up to 10 scheduled days within the first two months (0, 2, 6, 13, 20, 27, 34, 41, 48, and 59 days). They obtained information on newborns’ vital status, illness, and care practices during each visit [23,24].

### Data quality assurance

CHWs received training on recognition of infections, which consisted of screening newborns for ‘very severe disease’ or ‘suspected serious infection’, compared to the ‘gold standard’ (i.e., physicians’ assessment). The data collection procedure followed similar protocols conducted in training of health workers in the same study site [27]. Field supervisors directly supervised and ensured the quality of data collected by CHWs. To ensure the quality of newborns’ assessment and data collection, routine supervisory visits and standardized exercise sessions were organized in regular intervals. Additionally, the data entry system was custom-designed to check data consistency; in case of an inconsistency or incompleteness, CHWs conducted field verification to resolve the issue [27].

### Conceptual framework and study variables

**Fig 1:**
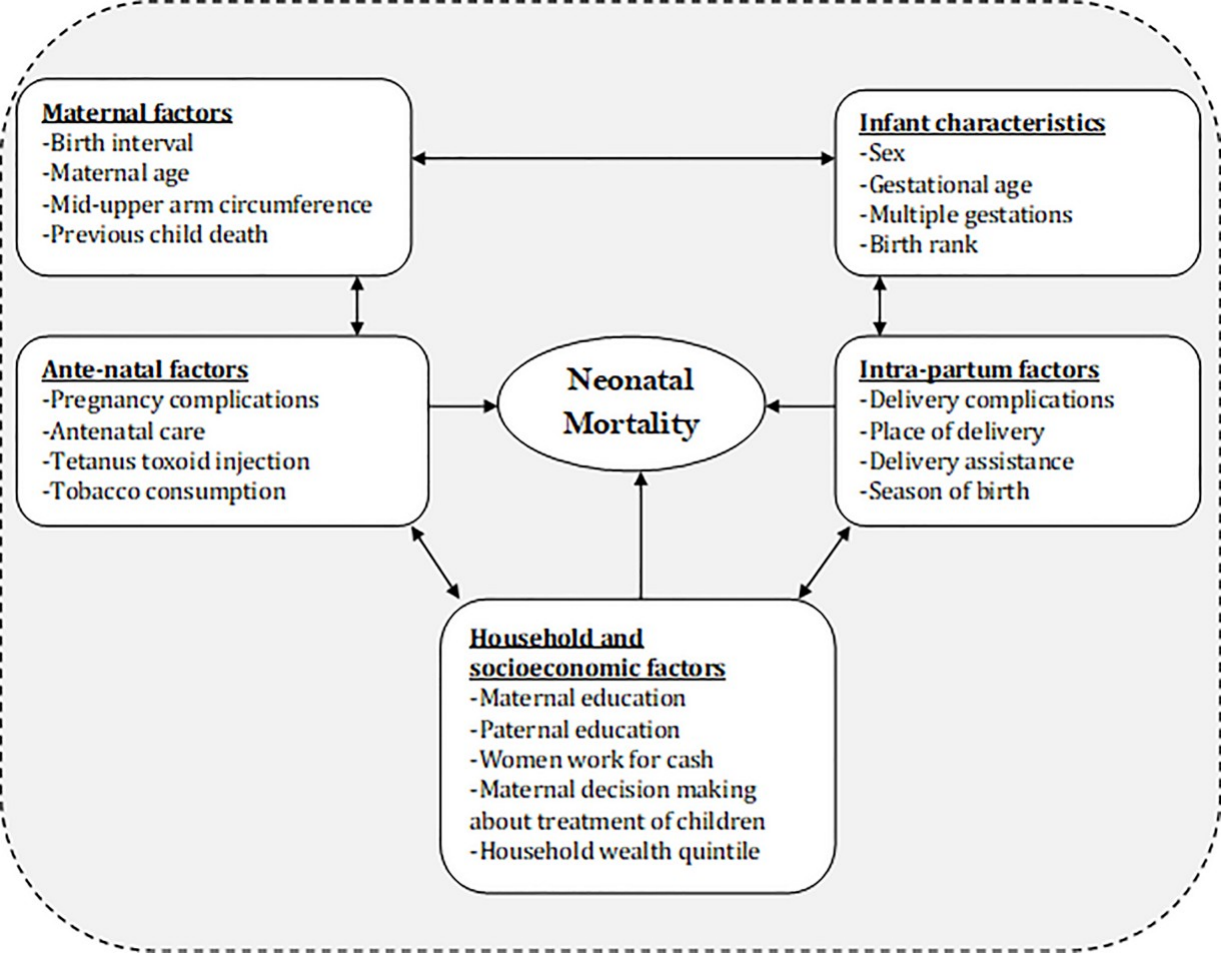
Conceptual framework for determinants of neonatal mortality *[adopted from Paudel et al*[28]*]*.

From the available theoretical frameworks to address childhood and neonatal mortality, we adopted a conceptual framework from a similar study conducted in Nepal by Paudel *et al*. [28]. This conceptual framework was adapted based on the data structure of the ANISA study. According to that framework, neonatal mortality is affected by the simultaneous presence of multiple factors. Potential risk factors for neonatal mortality were grouped into the following categories: (i) Infants’ individual characteristics: sex (male and female), gestational age (<34, 34–36, and ≥37 weeks), multiple gestations (yes and no), and birth rank (1^st^, 2^nd^ to 4^th^, and 5^th^ or higher); (ii) Maternal characteristics: birth interval (≤2 and 2–4 years), maternal age (<18, 18–24, 25–29, 30–34, and ≥35 years), maternal mid-upper arm circumference (MUAC) (<22 and ≥22 cm), history of previous child death (yes and no); (iii) Antenatal factors: antenatal complications (pregnancy complications such as high grade fever, excessive bleeding, convulsion, swelling of face or feet, foul smelling discharge or others), number of antenatal care seeking visits (no antenatal visit, 1–3, and ≥4 visits), receiving a tetanus toxoid injection (yes and no), and tobacco consumption during pregnancy (yes and no); (iv) Intrapartum factors: delivery complications such as excessive bleeding, convulsions, retained placenta, abnormal presentation, prolonged labor, premature water breaking, or others (yes and no), place of delivery (hospital and home delivery), delivery assistance (by skilled birth attendants, [doctors and nurses were included as skilled birth attendants]; yes and no), and season of delivery (winter or not winter); and finally (v) Socio-economic and household factors: maternal education (no formal education, primary, and secondary or above), women work for cash (yes and no), women’s decision-making power about treatment of children (yes and no), paternal education (no formal education, primary, and secondary or above), and household economic status (poorest, poorer, middle, richer, and richest) (Fig 1).

### Study participants

**Fig 2:**
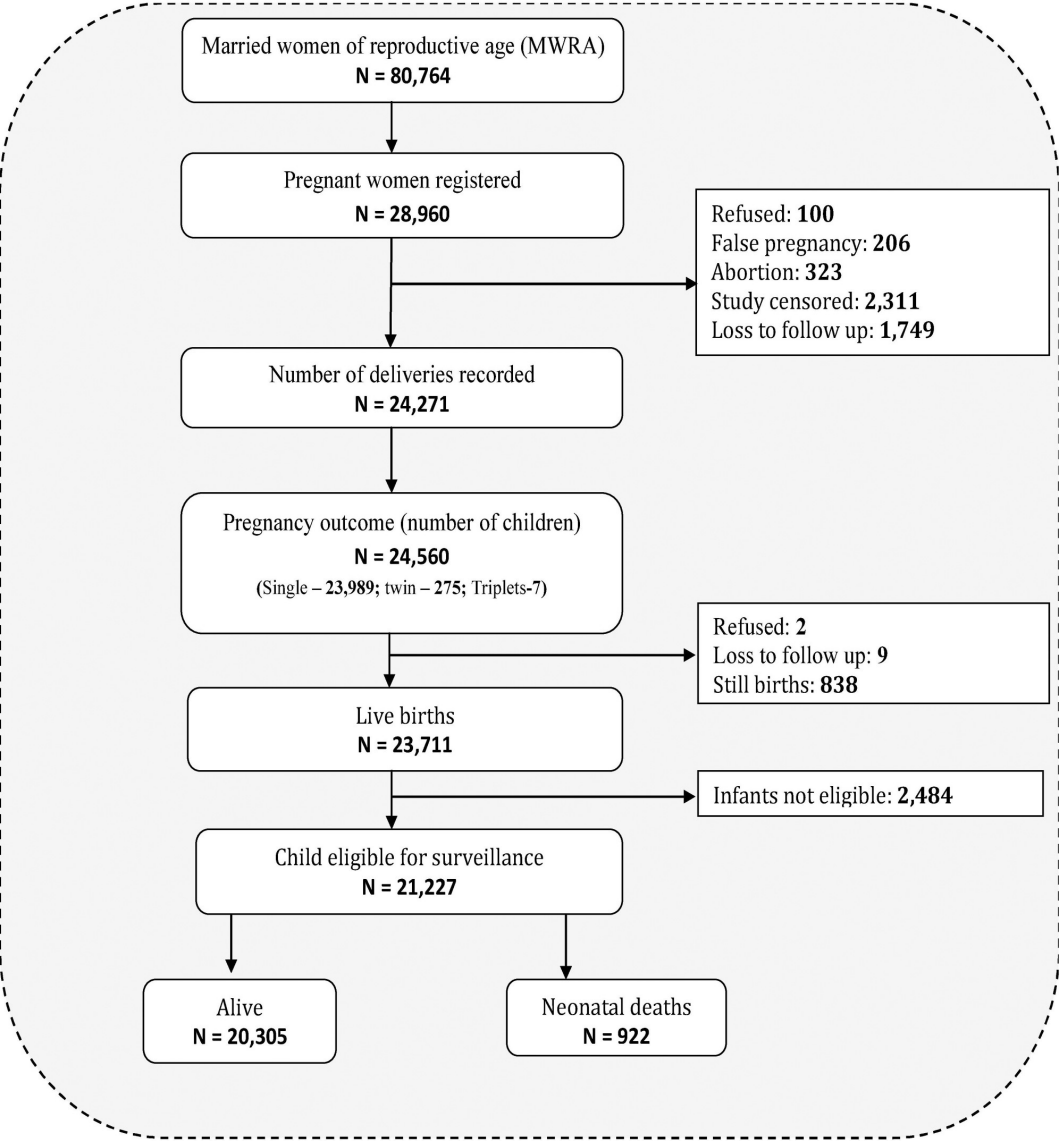
Study profile.

Fig 2 shows the study profile. Among 28,960 identified pregnant women, 4,689 (16.2%) women were excluded due to refusal (100), false pregnancy (206), abortion (323), administrative censoring before the occurrence of pregnancy termination (2,311), and loss to follow-up (1,749). Of the 24,271 recorded deliveries, 24,560 babies were born including 23,989 singletons, 275 twins, and 7 triplets. After excluding 838 stillbirths, 2 refusals, and 9 babies who were lost to follow-up, the total number of live births was 23,711. As 2,484 newborns were not eligible for follow-up due to the late identification, 21,227 newborns were included in the final analysis.

### Statistical analysis

Neonatal mortality was the dependent variable. This binary variable was coded as 0 for ‘no’ and 1 for ‘yes’. The NMR was calculated by dividing the number of newborns (n) who died within the first four weeks by the total number of included live-born babies (N). Newborns not enrolled within the first seven days after birth were not eligible for the ANISA surveillance and were excluded from analysis (Fig 2).

The principal component analysis of basic household materials (i.e., materials used to construct the walls, roof, and floor of the houses), drinking water source, sanitation facilities, availability of electricity, and household belongings was employed to construct wealth index for each household. The household wealth status was stratified into quintiles [29]. The percentage of missing data by variable was: maternal age (0.1%), gestational age (1.2%), and maternal mid-upper arm circumference (8.1%). The hot-deck method was used to impute missing data. In this procedure, other observations of the sample that have analogous characteristics generate the values for the missing observations [30]. Variance inflation factors (VIFs) were assessed to investigate multi-collinearity among explanatory variables. Two variables were correlated: ‘place of delivery’ and ‘delivery assistance’. Among these two collinear covariates, only ‘place of delivery’ was kept in the multivariable analysis. Continuous (e.g., maternal age) and discrete variables (e.g., parity) were converted into categorical variables.

Logistic regression was conducted to calculate unadjusted (i.e., crude) and adjusted odds ratios (ORs) and 95% confidence intervals (CIs). Variables with a predetermined significance level (p<0.2) in bivariate analyses were included in multivariable models. This significance level was sufficient to prevent residual confounding in multivariable models [31]. To observe how factors from various levels such as individual, maternal or household levels impact neonatal deaths, a three-step procedure was used to construct multivariable models. In Model 1, infants’ individual characteristics were included. In Model 2, maternal and intrapartum characteristics were added to the first model. In the final model, household & socioeconomic factors were included with Model 2. The best model was selected by obtaining log-likelihood, Akaike’s information criterion (AIC), adjusted-R^2^, and likelihood ratio tests. During logistic regression, we accounted for clustering among mothers who lived within the same union. Explanatory variables of each risk factor group have been shown in Fig 1. Stata 14.0 (Stata Corp, College Station, TX) was used to analyze data.

### Ethical approval

Ethical approval for the study protocol, consent forms (verbal and written), and data collection forms of the ANISA study was obtained from the institutional review board (IRB) of the Johns Hopkins Bloomberg School of Public Health (IRB No: 3,151) and the ethical review committee of the International Centre for Diarrhoeal Disease Research, Bangladesh (IRB No: 3,151).

## Results

Table 1 shows the NMR according to newborns’ background characteristics. Of the total 21,227 included newborns, 922 babies died within the neonatal period. The overall NMR was 43.4 (95% CI: 39.3–48.0) per 1,000 live births.

**Table 1:** Distribution of individual, maternal and socioeconomic characteristics along with NMR (95% CI).

Variables	(N = 21,227)	Neonatal deaths	
		n	NMR (95% CI)
**Infants’ individual characteristics**			
**Sex**			
**Male**	10,845 (51.1)	548	51.0 (45.5–56.1)
**Female**	10,382 (48.9)	374	36.0 (30.6–42.3)
**Gestational age (weeks)**			
**<34**	1,424 (6.7)	248	174.3 (147.6–204.1)
**34–36**	2,762 (13.0)	133	48.4 (33.8–66.5)
**≥37**	17,041 (80.3)	541	31.7 (28.3–35.8)
**Multiple gestation**			
**Yes**	441 (2.1)	125	283.4 (186.5–405.6)
**No**	20,786 (97.9)	797	38.3 (34.4–42.7)
**Birth rank**			
**First birth**	6,380 (30.0)	430	67.3 (60.2–75.3)
**2^nd^ to 4^th^ order**	10,916 (51.4)	329	30.1 (24.7–36.7)
**5^th^ or higher**	3,931 (18.6)	163	41.5 (34.8–49.4)
**Maternal and intrapartum factors**			
**Birth interval (between subsequent pregnancies)**			
**≤2 years**	2,931 (19.7)	106	36.2 (29.5–44.3)
**2–4 years**	11,916 (80.3)	386	32.4 (28.0–37.5)
**Maternal age (years)**			
**<18**	486 (2.3)	33	67.9 (39.1–115.2)
**18–24**	8,774 (41.3)	428	48.8 (42.5–55.9)
**25–29**	5,272 (24.8)	166	31.5 (25.7–38.4)
**30–34**	4,985 (23.5)	217	43.5 (35.4–53.3)
**≥35**	1,710 (8.1)	78	45.6 (40.9–50.8)
**History of previous child death**			
**Yes**	3,241 (15.3)	170	52.5 (43.0–63.8)
**No**	11,606 (54.7)	322	27.7 (23.6–32.6)
**Mid-upper arm circumference**			
**<22cm**	4,780 (22.5)	224	46.7 (36.4–59.6)
**≥22cm**	16,447 (77.5)	698	42.4 (38.1–47.3)
**Complications during pregnancy**			
**Yes**	3,943 (18.6)	248	62.9 (51.3–76.9)
**No**	17,284 (81.4)	674	39.0 (34.0–44.6)
**Antenatal care by a skilled provider**			
**No**	7,199 (33.9)	320	44.5 (36.9–53.4)
**1–3 visits**	9,744 (45.9)	423	43.4 (35.3–52.7)
**≥4 visits**	4,284 (20.2)	179	41.8 (36.9–48.6)
**TT-Injection during pregnancy**			
**Yes (≥1 TT injection)**	6,642 (31.3)	314	47.3 (40.1–55.6)
**No**	14,585 (68.7)	608	41.7 (37.2–46.7)
**Tobacco consumption during pregnancy**			
**Yes**	3,270 (15.4)	171	52.3 (43.6–62.7)
**No**	17,957 (84.6)	751	41.8 (37.9–46.1)
**Delivery complications**			
**Yes**	4,501 (21.2)	357	79.3 (70.7–88.8)
**No**	16,726 (78.8)	565	33.8 (29.2–39.0)
**Place of delivery**			
**Home**	18,734 (88.3)	744	39.7 (35.1–44.8)
**Hospital**	2,493 (11.7)	178	71.3 (60.1–84.7)
**Delivery assistance**			
**Unskilled attendants**	18,381 (86.6)	729	39.7 (34.2–45.9)
**Skilled attendants**	2,848 (13.4)	193	67.7 (56.4–81.3)
**Season of birth**			
**Not winter (Mar—Oct)**	12,731 (60.0)	572	44.9 (38.3–52.6)
**Winter (Nov—Feb)**	8,496 (40.0)	350	41.2 (37.0–45.8)
**Household and socio-economic Factors**			
**Maternal education**			
**No formal education**	5,162 (24.3)	253	49.0 (40.5–59.2)
**Primary**	7,933 (37.4)	363	45.8 (38.5–54.3)
**Secondary or higher**	8,132 (38.3)	306	37.6 (30.3–46.7)
**Paternal education**			
**No formal education**	7,590 (35.8)	355	46.8 (39.6–55.1)
**Primary**	7,389 (34.8)	326	44.1 (36.3–53.5)
**Secondary or higher**	6,248 (29.4)	241	38.6 (33.2–44.8)
**Women work for cash**			
**Yes**	449 (2.1)	20	44.5 (7.3–227.4)
**No**	20,778 (97.9)	902	43.4 (38.8–48.5)
**Women’s decision-making ability about** **children’s treatment**			
**Yes**	459 (2.2)	16	34.9 (14.6–80.9)
**No**	20,768 (97.8)	906	43.6 (39.5–48.2)
**Wealth quintile**			
**Poorest**	4,762 (22.4)	245	51.4 (43.3–61.0)
**Poorer**	3,757 (17.7)	177	47.1 (36.7–60.3)
**Middle**	4,281 (20.2)	184	43.0 (35.2–52.3)
**Richer**	4,198 (19.8)	167	39.8 (32.0–49.4)
**Richest**	4,229 (19.9)	149	35.2 (27.5–45.0)

NMR: Neonatal mortality rate, CI: Confidence interval; TT: Tetanus Toxoid

### Infants’ characteristics

About 51.1% of the included babies were males (n = 10,845). Male babies had a higher NMR compared to their female counterparts, 51.0 (95% CI: 45.5–56.1) and 36.0 (95% CI: 30.6–42.3) per 1,000 live births, respectively. The overall percentage of preterm births (i.e., <37 weeks of gestation) was 19.7%. Although only 6.7% (n = 1,424) of babies were born before 34 weeks of gestation, their NMR was high, 174 (95% CI: 147.6–202.9) per 1,000 live births. Likewise, babies born of multiple gestations had a higher NMR, 283.4 (95% CI: 186.5–405.6) per 1,000 live births. Both first-born and fifth or higher birth orders had higher NMR than the second to fourth birth order, 67.3 (95% CI: 60.2–75.3), 41.5 (95% CI: 34.8–49.4) and 30.1 (95% CI: 24.7–36.7) per 1,000 live births, respectively.

### Maternal and intrapartum characteristics

Compared to neonates born to 25–29 year-old-mothers, NMR among newborns born to younger (i.e., <25 years) or older (i.e., >29 years) mothers was higher with the highest NMR among the babies delivered by mothers <18 years of age, 67.9 (95% CI: 39.1–115.2) per 1,000 live births. Approximately two-thirds of the mothers (66.1%) were checked by medically trained providers during the pregnancy period. Maternal tobacco consumption during the pregnancy period resulted in a higher NMR compared to babies delivered by mothers without any tobacco consumption, 52.3 (95% CI: 43.6–62.7) and 41.8 (95% CI: 37.9–46.1) per 1,000 live births, respectively. A child delivered by a mother with a previous history of child death had an NMR of 52.5 (95% CI: 43.0–63.8) per 1,000 live births; this NMR was higher than a child delivered by a mother without any history of child death, 27.7 (95% CI: 23.6–32.6) per 1,000 live births. Children born to mothers with complications during childbirth had more than 1.5 times higher rate of dying compared to those without any complications, 62.9 (95% CI: 51.3–76.9) and 39.0 (95% CI: 34.0–44.6) per 1,000 live births, respectively. Most of the neonates were delivered at home (88.3%) or by unskilled attendants (86.8%). Babies born at hospitals or by skilled attendants had comparable NMR, 71.3 (60.1–84.7) and 67.3 (56.4–81.3) per 1,000 live births, respectively.

### Socioeconomic and household characteristics

More than three-fourths (75.7%) of the mothers and nearly two-thirds (64.2%) of the fathers received any formal education (i.e., attended school, college or madrasha). Newborns who had either a mother or father without any formal education had a higher NMR compared to having a mother or father with ‘secondary or above’ education level, with NMRs of 49.0 (95% CI: 40.5–59.2), 46.8 (95% CI: 39.6–55.1), 37.6 (95% CI: 30.3–46.7), and 38.6 (95% CI: 33.2–44.8) per 1,000 live births, respectively. Only 2.2% of the mothers had decision-making ability about their children’s treatment. Households’ wealth status showed an inverse pattern with the NMR. The NMR was highest among newborns from the lowest wealth quintiles and lowest among newborns from the highest wealth quintiles, 51.4 (95% CI: 43.3–61.0) and 35.2 (95% CI: 27.5–45.0) per 1,000 live births, respectively.

### Results of logistic regression analysis

Table 2 shows the results of simple and multivariable logistic regression analyses along with the model fit statistics. The first model (adjusted for individual factors) had all significant variables. Direction and magnitude of individual factors from the unadjusted level did not change significantly in this model. In Model 2, after adding the maternal & intrapartum characteristics, individual variables did not change significantly from Model 1. The association of maternal age with neonatal mortality attenuated, but one age group remained significantly associated: 30–34 years (AOR: 1.5, 95% CI: 1.2–1.9). Delivery complications (AOR: 2.0, 95% CI: 1.6–2.6) also had greater odds of neonatal deaths.

**Table 2:** Multivariable models for potential risk factors for neonatal mortality.

Variables	UOR	Model 1 Infants’ individual characteristics	Model 2 Maternal & intrapartum characteristics added	Model 3 Household & socioeconomic factors added
		AOR (95% CI)	AOR (95% CI)	AOR (95% CI)
**Infants’ characteristics**				
**Sex**				
**Female**	Ref.	Ref.	Ref.	Ref.
**Male**	1.4** (1.2, 1.7)	1.4*** (1.2, 1.7)	1.4** (1.1, 1.7)	1.4** (1.2, 1.7)
**Gestational age (weeks)**				
**<34**	6.4*** (5.2, 8.0)	5.5*** (4.3, 6.9)	5.5*** (4.3, 7.0)	5.3*** (4.2, 6.7)
**34–36**	1.5* (1.0, 2.3)	1.4^1^ (1.0, 2.0)	1.4^1^ (1.0, 2.0)	1.4^1^ (1.0, 1.9)
**≥37**	Ref.	Ref.	Ref.	Ref.
**Multiple gestation**				
**Yes**	9.9***(5.7, 17.4)	7.2*** (3.9, 13.5)	6.4*** (3.3, 12.4)	6.4*** (3.4, 12.1)
**No**	Ref.	Ref.	Ref.	Ref.
**Birth rank**				
**First birth**	1.7***(1.4, 2.0)	1.9*** (1.5, 2.4)	2.9*** (1.7, 5.3)	3.2*** (1.7, 5.9)
**2**^**nd**^ **to 4**^**th**^	0.7*(0.5, 1.0)	0.8^1^ (0.5, 1.1)	1.1 (0.6, 1.9)	1.2 (0.7, 2.1)
**≥5**	Ref.	Ref.	Ref.	Ref.
**Maternal and intrapartum characteristics**				
**Birth interval (years)**				
**≤2**	1.1 (0.8, 1.5)			
**2–4**	Ref.			
**Maternal age (years)**				
**<18**	2.2** (1.3, 3.7)		1.4 (0.7, 2.8)	1.4 (0.7, 2.9)
**18–24.9**	1.6*** (1.3, 1.9)		1.1 (0.8, 1.6)	1.2 (0.9, 1.5)
**25–29.9**	Ref.		Ref.	Ref.
**30–34.9**	1.4** (1.1, 1.8)		1.5* (1.1, 1.9)	1.4* (1.1, 1.9)
**≥35**	1.5**(1.2, 1.8)		1.4* (1.0, 2.0)	1.4^1^ (1.0, 1.9)
**History of child death**				
**Yes**	1.9*** (1.4, 2.6)		1.7** (1.2, 2.3)	1.6** (1.2, 2.2)
**No**	Ref.		Ref.	Ref.
**Mid-upper arm circumference**				
**<22 cm**	1.1 (0.8, 1.5)			
**≥22 cm**	Ref.			
**Complications during pregnancy**				
**Yes**	1.6*** (1.2, 2.2)		1.3^1^ (0.9, 1.8)	1.3^1^ (0.9, 1.8)
**No**	Ref.		Ref.	Ref.
**Antenatal care by a skilled provider**				
**No**	1.0 (0.8, 1.4)			
**1–3 visits**	1.0 (0.8, 1.3)			
**≥4 visits**	Ref.			
**TT-Injection during pregnancy**				
**Yes (≥1 TT injection)**	1.1 (0.9, 1.4)		1.0 (0.7, 1.3)	1.0 (0.8,1.3)
**No**	Ref.		Ref.	Ref.
**Maternal tobacco consumption during pregnancy**				
**Yes**	1.3** (1.1, 1.5)		1.3 (1.0, 1.6)	1.2^1^ (1.0, 1.4)
**No**	Ref.		Ref.	Ref.
**Delivery complications**				
**Yes**	2.5*** (2.0, 3.0)		2.0*** (1.6, 2.6)	2.0*** (1.6, 2.6)
**No**	Ref.		Ref.	Ref.
**Place of delivery**				
**Facility delivery**	1.9*** (1.5, 2.4)		1.0 (0.8, 1.4)	1.1 (0.8, 1.5)
**Home delivery**	Ref.		Ref.	Ref.
**Delivery assistance**				
**Unskilled attendants**	0.6*** (0.4, 0.8)			
**Skilled attendants**	Ref.			
**Season of birth**				
**Not winter (Mar-Oct)**	1.1 (0.9, 1.3)			
**Winter (Nov-Feb)**	Ref.			
**Household and socioeconomic Factors**				
**Maternal education**				
**No formal education**	1.3^1^ (0.9, 1.9)			1.3 (0.7, 2.4)
**Primary**	1.2^1^ (0.9, 1.7)			1.2 (0.8, 1.9)
**Secondary or higher**	Ref.			Ref.
**Paternal education**				
**No formal education**	1.2^1^ (0.9, 1.6)			1.0 (0.7, 1.3)
**Primary**	1.1^1^(0.9, 1.4)			1.0 (0.8, 1.2)
**Secondary or higher**	Ref.			Ref.
**Women work for cash**				
**Yes**	1.0 (0.2, 6.8)			
**No**	Ref.			
**Women’s decision-making ability about children’s treatment**				
**Yes**	0.8 (0.3, 1.9)			
**No**	Ref.			
**Wealth quintile**				
**Poorest**	1.5*** (1.1, 2.0)			1.4 (0.9, 2.3)
**Poorer**	1.4* (0.9, 1.9)			1.2 (0.8, 2.0)
**Middle**	1.2 (0.9, 1.7)			1.2 (0.8, 1.6)
**Richer**	1.1 (0.8, 1.6)			1.2 (0.8, 1.8)
**Richest**	Ref.			Ref.
**Log-likelihood**	-	-3406	-3327	-3319
**AIC**	-	6824	6691	6677
**Adjusted-R**^**2**^	-	0.0493	0.0622	0.0625
**Average VIF**	-	1.01	1.11	1.10

1 p<0.2

* p<0.05

** p<0.01

*** p<0.001

UOR: Unadjusted odds ratio, AOR: Adjusted odds ratio, AIC: Akaike Information Criterion, TT: Tetanus Toxoid VIF: variance inflation factor

After adding the household and socioeconomic factors in Model 3, some variables remained significantly associated with the outcome variable. Compared to singleton babies, the odds of neonatal mortality were more than six times higher among babies born to mothers with multiple gestations (AOR: 6.5, 95% CI: 3.3–12.5). Babies born with gestational age <34 weeks also had higher odds compared to term (i.e., ≥37 weeks) babies (AOR: 5.3, 95% CI: 4.2–6.8). Ordered from the most significant associated factors, neonates with the following characteristics had significantly increased odds of dying: first birth (AOR of 2^nd^ to 4^th^ child vs first birth order: 3.0, 95% CI: 1.7–5.4), delivery complications (AOR: 2.1, 95% CI: 1.6–2.7), history of previous child death (AOR: 1.6, 95% CI: 1.2–2.2), and male sex (AOR: 1.4; 95% CI: 1.2–1.7). Although parental education level and household wealth status were associated with NMR in bivariate analyses, none of these factors were significantly associated in the final multivariable model.

The log-likelihood of neonatal mortality increased from Model 1 to Model 3. On the other hand, the AIC decreased from Model 1 to Model 3. The average VIF of Model 3 was 1.10. These model fit statistics including the adjusted-R^2^ indicate that Model 3 explains the variability better than other models.

## Discussion

In this community-based cohort study, the burden of neonatal mortality was high at the rate of 43.4 per 1,000 live births. The following factors were associated with higher rate and odds neonatal mortality: male sex, multiple gestations, lower gestational age, first-born child, maternal age of ≥30 years, and a history of antenatal and delivery complications. Although some characteristics such as parental education level and household wealth status were significantly associated with NMR in crude analyses, the adjusted association was not significant for these factors.

The NMR observed in this study was relatively higher than the national average (28 per 1,000 live births) as well as the overall rate in Sylhet Division (39 per 1,000 live births) [3]. This study was conducted in rural regions of Sylhet Division; in addition to a high NMR in Sylhet division, the overall NMR in rural regions is usually higher than the overall rate in Bangladesh. The high NMR we observed could be explained by the fact that this study was conducted in a rural region in the division with the highest NMR in this country. Furthermore, rural women have poor access to health care [3,32], and all maternal and child health indicators are poorer in Sylhet Division compared to other divisions in Bangladesh [2,3].

We found that nearly one-fifth of the babies were preterm, similar to findings from a previous study conducted in this site [33]. Consistent with earlier reports, preterm babies had higher rates and odds of neonatal deaths [9,10,28,34]. Preterm babies have increased risks of infections, hypoglycemia, and hypothermia due to physical immaturity [35]. Globally, prematurity is a leading cause of neonatal deaths [36]. To reduce this high NMR in Bangladesh, the prevention and management of prematurity will be crucial. Several behavioral (e.g., smoking cessation) and medical interventions (e.g., progesterone supplementation) could prevent preterm births [37]. KMC is a useful and low-cost intervention for these babies, as most of the children are born at home without skilled attendants [38,39]. Similar to other studies, we have found an association between neonatal mortality and multiple gestations; this is a well-known risk factor for preterm birth [33,40]. Babies delivered by mothers with multiple pregnancies have greater risks of preterm deliveries as well as antenatal and delivery complications. Simultaneous presence of these factors contributes to higher number of deaths [33,40]. These mothers require adequate birth preparedness to prevent neonatal deaths.

Male neonates had higher rates and odds of dying compared to their female counterparts as has been reported previously [33,41,42]. Male newborns are also more susceptible to infections due to immunodeficiency [43] and have an increased likelihood of congenital malformations [44]. These conditions could increase the risk of death among male newborns. Although the sex of a baby is unmodifiable, this factor is significant from a program planning perspective, in that male infants may require greater attention.

Neonates from mothers with delivery complications such as vaginal bleeding, convulsion or prolonged labor were more likely to die than the neonates born to women without these complications. This finding is common among studies that have examined risk factors for neonate mortality, and indicates that appropriate management of delivery complications would be crucial to ensure better survival of neonates and reduce the overall NMR [12,45–48].

We found an association of birth order with neonatal mortality; first-born children were more likely to die than their next siblings [49]. Prior studies have demonstrated a U-shaped relationship between birth order and neonatal mortality [50]. Mothers of premature infants may not be able to handle sickness of the babies due to inexperience or inability to understand the new responsibilities of child care [11]. Though we did not find teen pregnancy as a risk factor after adjusting for other factors, birth order has been linked with teen pregnancy [11,49]. A significant proportion of primi infants are born to mothers who became pregnant during their teenage years. Along with being inexperienced [11], these mothers perhaps give birth to premature babies because they do not reach full physical or reproductive maturity before pregnancy [49]. There is a need to implement interventions to delay first pregnancy and antenatal interventions to educate first-time mothers.

Our results showed that neonates were more likely to die with a maternal age above 30 years. Consistent with our study findings, a previous study from Bangladesh also found an association between maternal age and neonatal deaths [10]. Parity and maternal age are correlated, as an older mother would have a higher parity than a younger mother, and a mother with a high parity would have higher age compared to a mother with low parity. Earlier studies have observed that neonatal mortality is associated with 5^th^ or higher birth rank [12,34]. As this is a modifiable factor associated with neonatal mortality, these mothers would require appropriate birth preparedness to ensure neonatal survival.

Women who had previously endured the death of a child were more likely to also experience a death of their newborns in the neonatal period, a finding similarly observed in a case-control study in Bangladesh [51]. Adequate birth preparedness could ensure better neonatal survival among this high-risk group.

Most newborns were delivered at home or by unskilled attendants. Newborns born in health facilities or by skilled attendants had higher NMR compared to newborns born at home or by unskilled attendants. Although this was not a significant factor in multivariable analysis, the higher NMR among these groups could result from complications during childbirth when women were more likely to be delivered in health facilities or by skilled attendants. The mothers utilized these services as ‘curative’ (i.e., to manage delivery complications) services instead of regular or preventive services [26,45].

This study found no association between neonatal mortality and socioeconomic factors; although household wealth status showed a significant association in unadjusted analysis, it was not significant after adjustment. This finding is contrary to those of similar studies from Bangladesh [9,10,20] or other countries [11,12]. However, a recent analysis by McKinnon et al. concluded that the inequalities associated with neonatal mortality are declining in developing countries including Bangladesh, and thus our results are consistent with that finding [52]. Implementation of maternal and neonatal survival programs helped to overcome the socioeconomic barriers of neonatal deaths in these countries [52–54]. The insignificance of the association between socioeconomic and many other factors and NMR indicates that these variables mainly influence neonatal deaths through other intervening factors that were significant such as maternal age, birth order, previous child death, multiple gestations and preterm births. To reduce neonatal deaths, addressing these maternal and intrapartum factors would be important.

Our study has several notable strengths. We analyzed prospectively collected data; recall bias was minimal for pregnancy and delivery complications. Recall bias is a problem of studies which analyze cross-sectional demographic and health survey (DHS) data [9,10,20]. Given the cohort design, we were able to collect data of the women who died due to delivery-related complications while studies that analyzed DHS data could not analyze data of women who die due to pregnancy-related complications; maternal death is closely associated with neonatal death. Our results are generalizable to other rural areas in Bangladesh or in other developing countries. The sample size was large to provide precise estimates. The proportion of missing data was small.

One limitation of our study is that we were unable to include all registered pregnant women (16.2%) or delivered babies (13.6%). Some women were absent on repeated visits, while other women out-migrated during the study period or refused to participate. However, the main reason for not including women in the analysis was that these women did not have a pregnancy outcome during the study follow-up period. Though, these women were unlikely to be different from women who completed pregnancy. Only newborns visited by a CHW within seven days after birth were eligible for ANISA surveillance. Since neonatal deaths occur at a higher rate in the first seven days of life, this exclusion might have led to an underestimation of the burden of neonatal mortality. We relied on the last menstrual period’s (LMP) date to calculate gestational age; it could have led to misclassification of preterm births due to possible recall errors of LMP [33]. Due to lack of data, we could not investigate several other known determinants of neonatal mortality including environmental and genetic factors [11,12].

## Conclusion

Our study investigated rates and risk factors for neonatal mortality in rural regions of the division with the highest risk of neonatal deaths in Bangladesh. We found several individual (e.g., sex of the baby), maternal (e.g., maternal age), and intrapartum (e.g., delivery complications) factors that have relationships with higher rates and likelihoods of neonatal mortality. Simultaneous impacts of these factors on neonatal mortality indicate that a comprehensive strategy is required to address them. Prioritizing modifiable risk factors through community-based programs is essential to achieve the NMR target of the SDGs; intrapartum factors are important for this prioritization. Programs need to address reducing the number of preterm births. KMC should be implemented at the community level to manage preterm babies. Adequate birth preparedness is required for ‘primi’ mothers, mothers with multiple pregnancy and with a history of child death. Timely referral and management of antenatal and delivery complications should be ensured. Lastly, more community-based studies are required to determine the feasibility of above-mentioned interventions in rural settings.

## Abbreviations

AIC: Akaike information criterion
ANISA: Aetiology of Neonatal Infections in South Asia
AOR: Adjusted odds ratio
CHW: Community Health Worker
CI: Confidence interval
DHS: Demographic and Health Survey
LMP: Last menstrual period
MDGs: Millennium Development Goals
MUAC: Mid-upper arm circumference
NMR: Neonatal Mortality Rate
Projahnmo: Project for Advancing Health of Newborns and Mothers
SDGs: Sustainable Development Goals
UOR: Unadjusted odds ratio
VIF: Variance Inflation Factor
WHO: World Health Organization
